# App-based symptom tracking to optimize SARS-CoV-2 testing strategy using machine learning

**DOI:** 10.1371/journal.pone.0248920

**Published:** 2021-03-25

**Authors:** Leila F. Dantas, Igor T. Peres, Leonardo S. L. Bastos, Janaina F. Marchesi, Guilherme F. G. de Souza, João Gabriel M. Gelli, Fernanda A. Baião, Paula Maçaira, Silvio Hamacher, Fernando A. Bozza

**Affiliations:** 1 Department of Industrial Engineering, Pontifical Catholic University of Rio de Janeiro, Rio de Janeiro, RJ, Brazil; 2 Instituto Tecgraf, Pontifical Catholic University of Rio de Janeiro, Rio de Janeiro, RJ, Brazil; 3 National Institute of Infectious Diseases Evandro Chagas (INI), Oswaldo Cruz Foundation (FIOCRUZ), Rio de Janeiro, RJ, Brazil; 4 D’Or Institute for Research and Education (IDOR), Rio de Janeiro, RJ, Brazil; University of Greenwich, UNITED KINGDOM

## Abstract

**Background:**

Tests are scarce resources, especially in low and middle-income countries, and the optimization of testing programs during a pandemic is critical for the effectiveness of the disease control. Hence, we aim to use the combination of symptoms to build a predictive model as a screening tool to identify people and areas with a higher risk of SARS-CoV-2 infection to be prioritized for testing.

**Materials and methods:**

We performed a retrospective analysis of individuals registered in "*Dados do Bem*," a Brazilian app-based symptom tracker. We applied machine learning techniques and provided a SARS-CoV-2 infection risk map of Rio de Janeiro city.

**Results:**

From April 28 to July 16, 2020, 337,435 individuals registered their symptoms through the app. Of these, 49,721 participants were tested for SARS-CoV-2 infection, being 5,888 (11.8%) positive. Among self-reported symptoms, loss of smell (OR[95%CI]: 4.6 [4.4–4.9]), fever (2.6 [2.5–2.8]), and shortness of breath (2.1 [1.6–2.7]) were independently associated with SARS-CoV-2 infection. Our final model obtained a competitive performance, with only 7% of false-negative users predicted as negatives (NPV = 0.93). The model was incorporated by the "*Dados do Bem*" app aiming to prioritize users for testing. We developed an external validation in the city of Rio de Janeiro. We found that the proportion of positive results increased significantly from 14.9% (before using our model) to 18.1% (after the model).

**Conclusions:**

Our results showed that the combination of symptoms might predict SARS-Cov-2 infection and, therefore, can be used as a tool by decision-makers to refine testing and disease control strategies.

## Introduction

The current COVID-19 pandemic caused by the SARS-CoV-2 requires extensive testing programs to understand the transmission, diagnose, and isolate the positive cases. Given the high mortality and absence of a specific treatment or a reliable vaccine, large testing programs are an essential part of epidemic control. The frequency of testing, however, is very heterogeneous among countries. Brazil currently has the second-highest number of COVID-19 cases, even with lower test rates (120,548 tests per one million inhabitants, as of December 02, 2020) [1], which makes screening systems essential to prioritize testing. In the past, some successful monitoring systems have already been introduced, such as the Influenzanet consortium, which enables monitoring the community in real-time and estimating risk factors for common diseases such as influenza [2].

Some screening tools also have already been introduced, aiming to predict the epidemic trend of COVID-19. Zhu et al. [3] proposed an online learning framework for public health emergency surveillance based on the heart rate and sleep data collected from wearable devices. The results showed that the predictive model could alert the infection outbreak in advance. Quer et al. [4] developed a smartphone app that collects self-reported symptoms, personal sensor data, and diagnostic testing results from individuals in the United States. They assessed the difference between COVID-19 positive versus negative cases in symptomatic individuals. Mehl et al. [5] analyzed the added value of a mobile phone app-based symptom assessment tool, known as Ada, that collects individual information and then guides them to the most appropriate care. Menni et al. [6] used information from an app-based symptom tracker from UK and USA. They concluded that the combination of symptoms could be used as a screening tool to identify people with a possible positive result for COVID-19. However, little is known about symptoms association and model potential usage as a screening tool in low- and medium-income countries (LMIC) such as Brazil.

Thus, our study aims to use the combination of symptoms and machine learning techniques to develop a predictive model that identifies people and areas with a higher risk of SARS-CoV-2 infection. We used data from an app-based symptom tracker known as "*Dados do Bem*" [7], which is an initiative that became available for the city of Rio de Janeiro, one of the centers of the outbreak in the country. With our model, we could estimate the proportion of infected participants and then categorize risk levels of infection prevalence within the geographical area of Rio de Janeiro. The results revealed that incorporating our model in the app increased the test results’ positivity rate and reached a higher seroprevalence than the city-level prevalence reported by Hallal et al. [8], thus showing an improvement of the testing strategy.

## Materials and methods

### Study design and data source

This study is a retrospective analysis of prospectively collected data from individuals registered in the "*Dados do Bem*" app. This large Brazilian initiative combines an app-based symptom tracker and a public testing initiative for the users. The app interface and the survey questions are provided in S1 Fig.

The free smartphone application was launched in Brazil on April 28, 2020. Through a short survey, it collects geo-referenced data from subscribed users, their demographic and occupational characteristics, self-reported symptoms, as well as whether the participant is a health professional and was in contact with a SARS-CoV-2 infected person. The app then combines the surveyed information and selects individuals for testing through selection criteria (see S1 File). Those indicated by a previously positively tested participant have the highest priority to be tested, followed by health professionals. The test used at the study time was the antibody WondfoCOVID-19 IgM/IgG test (sensitivity = 86.43%, specificity = 99.57%) [9], available only for Rio de Janeiro.

### Study population

We included participants registered through the smartphone app from its launch date until July 16, 2020. To train the model, we selected participants who responded to the questionnaire, made the antibody WondfoCOVID-19 IgM/IgG test in a location designated by the app within the city of Rio de Janeiro, and obtained a result (positive or negative). For identifying risk areas, we also included the participants that had not been tested, applying the model to estimate their test results.

### Outcomes and variables

Our primary outcome was the test result (positive or negative) at the user level. Our goal was to identify clinical manifestations and individual factors associated with positive testing. Hence, we collected and assessed participant demographics (age, gender), nine symptoms (loss of smell or anosmia, fever, myalgia, cough, nausea, shortness of breath, diarrhea, coryza, and sore throat), and whether the user lives together with someone with a confirmed SARS-CoV-2 infection.

### Statistical analysis

We described the characteristics and symptoms of positive and negative tested participants, displaying the mean and standard deviation for continuous variables and the frequency for categorical variables. We then analyzed the individual association between symptoms and the test result using a logistic regression model adjusted to age and gender. That is, we fitted 11 (one for each feature) logistic regression models, where the response variable was the test result, and the explanatory variables were age, gender, and each of the features. The intention was to remove the confounding effects of age and gender in analyzing the symptoms, obtaining an odds ratio with less interference. We provided the corresponding Odds Ratio (OR) with a 95% confidence interval.

We aim to identify a combination of symptoms to build a prediction model for determining a participant with SARS-CoV-2 infection. For that, we compared five different machine learning techniques: Logistic Regression (LR) stepwise, Naïve Bayes (NB), Random Forest (RF), Decision Tree using C5.0 (DT), and eXtreme gradient Boosting. To address the imbalanced response variable (only 11.8% are positive tests) during model training, we also evaluated four different data balancing techniques: Downsampling, Upsampling, Synthetic Minority Oversampling Technique (SMOTE) [10], and Random Over-Sampling Examples (ROSE) [11]. Definitions about these methods and balancing approaches can be seen in S2 File.

We divided the data into a training set (80%) and a testing set (20%), keeping the same proportion of majority and minority classes among subsamples. The training set creates predictive models, and the remaining validate the proposed model. During model training, for each combination of machine learning techniques and balancing strategies, we applied grid-search hyperparameter optimization with 5-fold cross-validation, using the Area Under the ROC Curve (AUC) as the target metric. It is independent of a specific cut-off value [12], which allows for a better evaluation of the model behavior during the training process.

After obtaining the best hyperparameters for each model, we applied Matthews Correlation Coefficient (MCC) to evaluate the results in the test sets since it is a balanced measure among True Positives, True Negatives, False Positives, and False Negatives, which are based on a preset score threshold [13]. The chosen cut-off point for predicted values was 50%, i.e., participants with a probability higher than 50% were classified as "positive," otherwise "negative" [14]. In addition to the MCC value, we considered the model intelligibility for choosing our final model.

Finally, we evaluated the distribution of SARS-CoV-2 infection risks over the geographic area of Rio de Janeiro modeled as a grid map (each grid is a 400m x 400m square area). Along with the participants with confirmed test results, we applied the chosen model to the sample of participants that were still untested in the period of this study to obtain their estimated test result. We then calculated the proportion of estimated SARS-CoV-2 infections for each grid according to Eq 1.

$$Grid\;risk=\frac{Number\;of\;positive\;users\;in\;the\;grid\;according\;to\;the\;model}{all\;grid\;users}$$ (1)

To avoid misinterpreting proportions in grids with scarce data, we considered grids with at least 10 participants (~94% of all observations). Then, we evaluated the distribution of the grid risks among all grids and classified them into five risk groups using the mean ± 0.5 and 1.5 standard deviations (SD) as thresholds: "very low" (< mean-1.5*SD), "low" (from mean-1.5*SD to mean-0.5*SD), "medium" (from mean-0.5*SD to mean+0.5*SD), "high" (from mean+0.5*SD to mean+1.5*SD), and "very high risk" (>mean+1.5*SD). Using this classification, we built a risk map for Rio de Janeiro.

All analyses were performed in R 3.6.3, using ’*tidyverse*’ package for data wrangling and plots; and ’*caret*’ for the prediction models, with *’glm’* for Logistic Regression, ’*ranger*’ for Random Forest, ’*C50*’ for Decision Trees, ’*xgbTree’* for the eXtreme gradient Boosting, and ’*naivebayes’* for the Naïve Bayes model. The code used for estimating the models is available in a Github repository (https://github.com/noispuc/Dantas_etal_PLOSOne_App-based-symptom).

### External validation design

Our final model was incorporated into the app on July 17, 2020. To verify the gains using this proposed model, we performed a validation using Rio de Janeiro’s data. We compared the proportion of positive results before the model was implemented in the app (using data from June 15, 2020, to July 16, 2020) and after its implementation (using data from August 01, 2020, to September 01, 2020). The two-week interval between incorporating the model into the app and the validation was necessary as there were still tests scheduled according to the previous prioritization policy.

We used the unpaired two-samples Wilcoxon test to investigate the hypothesis that the difference between the proportion of positive results before and after the model implementation is statistically significant, with a confidence level of 0.95. We evaluated if the mean of the proportions of positive results before implementing the model can be considered less than the average proportion of positive results after its implementation. Since this compares non-normally distributed data, the Wilcoxon test is the most appropriate hypothesis test.

### Ethics statement

The study is retrospective. All data acquired were anonymized, and the "*Dados do Bem*" app follows the Brazilian General Data Protection Regulation (*Lei Geral de Proteção de Dados*—LGPD). All users provided informed consent of de-identified data-use to non-commercial research upon registration in the app. All answers were optional.

## Results

### Characteristics and self-reported symptoms associated with SARS-CoV-2 infection

From April 28, 2020, to July 16, 2020, 337,435 individuals registered their symptoms through the smartphone app. Of these, 49,721 users were then tested, from which 5,888 (11.8%) received a positive result for SARS-CoV-2 infection.

**Fig 1:**
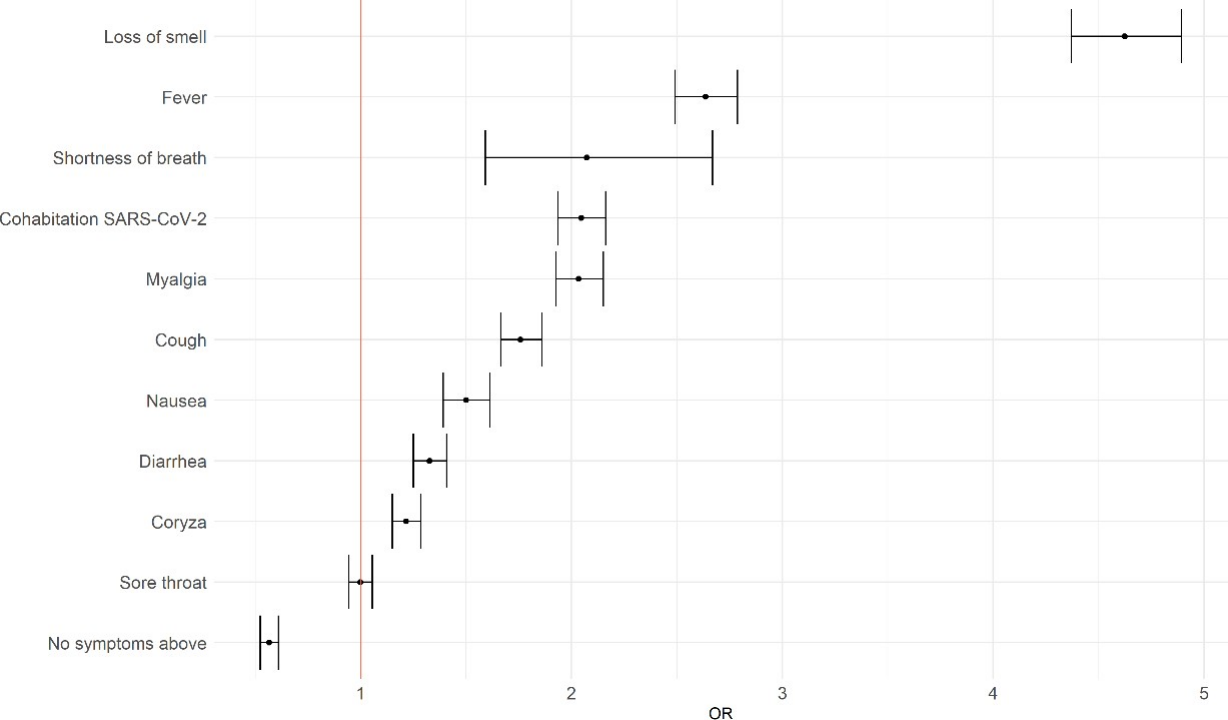
Association between symptoms and the SARS-CoV-2 infection. The Odds Ratio (OR) with 95% confidence intervals using logistic regression models for each feature was adjusted by age and gender.

According to the self-reported information (Table 1), most participants were women (61.9%), health professionals (55.8%), with a median age of 41 years old (IQR: 33–51). Among those who tested positive for SARS-CoV-2 infection, cough was the most frequent symptom (59.6%), followed by myalgia (57.4%), coryza (56.3%), loss of smell/anosmia (52.9%), and fever (44.8%). When evaluating the association between each symptom and the test result, adjusted for age and gender (Fig 1), we found a similar result: loss of smell (odds ratio [OR]: 4.6; 95% CI: 4.4–4.9), fever (OR: 2.6; 95% CI: 2.5–2.8), and shortness of breath (OR: 2.1; 95% CI: 1.6–2.7) were associated with a positive result for SARS-CoV-2 infection.

**Table 1:** Characteristics and symptoms of the study population tested for SARS-CoV-2 infection.

	Total	Positive test	Negative test
**Participants, n (%)**	49,721	5,888 (11.8)	43,833 (88.2)
**Characteristics**			
Female, n (%)	30,769 (61.9)	3,641 (61.8)	27,128 (61.9)
Age (years), median [IQR]	41 [33–51]	43 [34–53]	40 [33–51]
Cohabitation—lives with a SARS-CoV-2 infected person, n (%)	20,944 (42.1)	3,398 (57.7)	17,546 (40.0)
Health professional, n (%)	27,737 (55.8)	3,099 (52.6)	24,638 (56.2)
**Self-reported symptoms, n (%)**			
Coryza	25,973 (52.2)	3,315 (56.3)	22,658 (51.7)
Cough	23,430 (47.1)	3,507 (59.6)	19,923 (45.5)
Myalgia	20,858 (42.0)	3,380 (57.4)	17,478 (39.9)
Sore throat	20,794 (41.8)	2,459 (41.8)	18,335 (41.8)
Fever	13,042 (26.2)	2,640 (44.8)	10,402 (23.7)
Diarrhea	12,573 (25.3)	1,778 (30.2)	10,795 (24.6)
Loss of smell	11,835 (23.8)	3,112 (52.9)	8,723 (19.9)
Nausea	6,461 (13.0)	1,025 (17.4)	5,436 (12.4)
Shortness of breath	354 (0.7)	74 (1.3)	280 (0.6)
No symptoms above	10,865 (21.9)	844 (14.3)	10,021 (22.9)

Results are displayed in median (interquartile range, IQR) for continuous variables and percentage values for categorical variables.

### Combination of symptoms and predictive modeling

**Fig 2:**
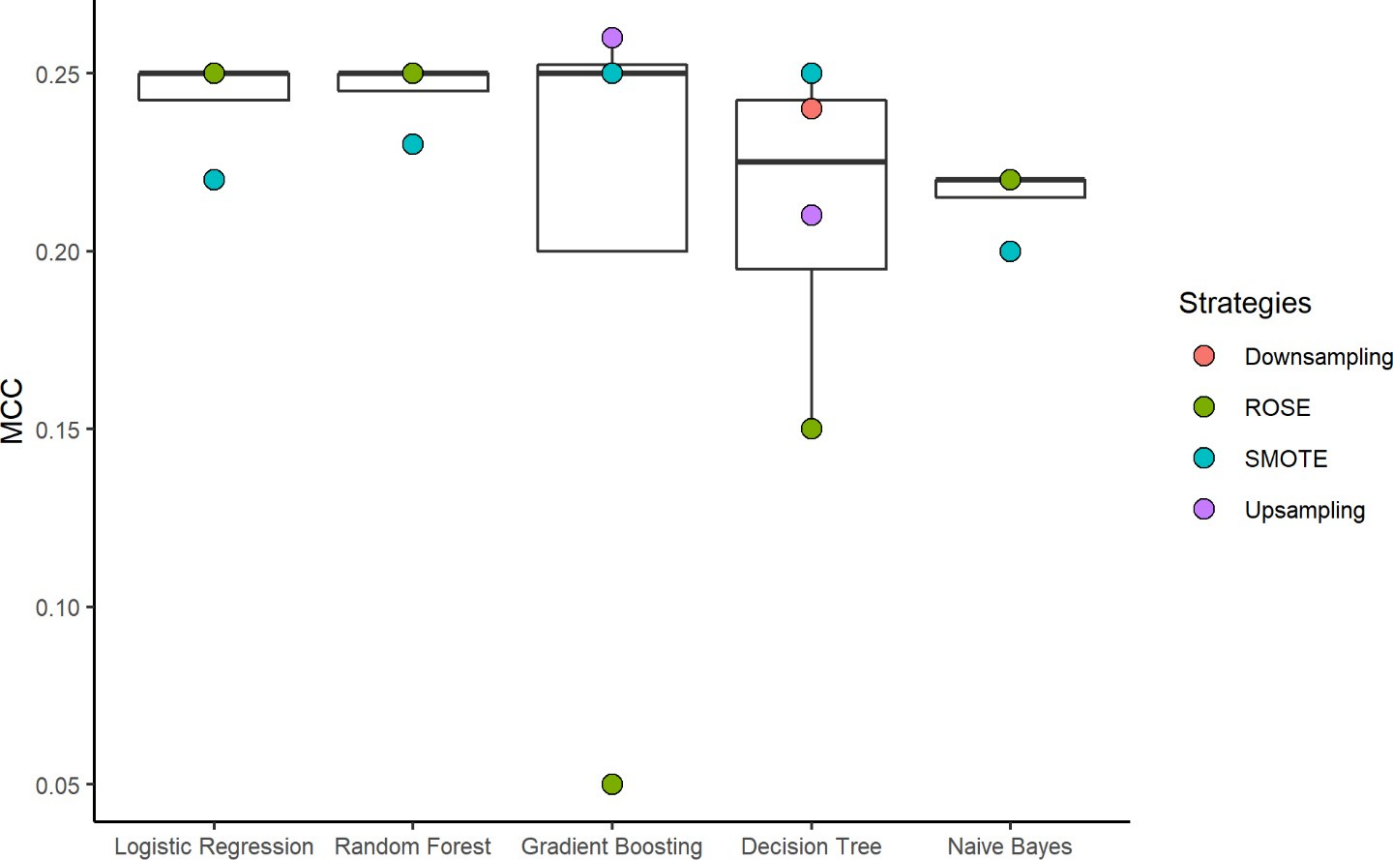
Boxplots representing the Matthews Correlation Coefficients (MCC) of each model and balancing technique combination (points) for all methods. Boxplots represent the distribution of MCC values for each model and balancing technique combination. The higher the MCC value, the better the model.

To develop a model to predict positive participants based on the available dataset, we ran 25 different combinations of machine learning techniques and sampling strategies. We comparatively evaluated the performance of the models on the test set according to the metrics of Sensitivity, Specificity, Predictive Positive Value (PPV), Negative Predictive Value (NPV), F1-Score, and MCC. The logistic regression, gradient boosting, and random forest techniques presented the best median MCCs, followed by the decision tree and naïve Bayes, as shown in Fig 2. The results of all combinations and metrics can be seen in the S1 Table.

According to Fig 2, the performance of the balancing strategies varied among the methods. SMOTE had the worst results in the LR, RF, and NB models, while downsampling, ROSE, and upsampling performed best in these models. On the other hand, SMOTE was the best in the decision tree model, while the upsampling approach obtained the worst results for this technique.

Our final model resulted from the logistic regression method combined with the upsampling balancing strategy (Eq 2). The logistic regression model was chosen since it is the most intelligible; that is, a single feature’s contribution to the final prediction can be easily understood in the model [15].

$$Probability\;of\;testing\;positive=\frac{e^{prediction}}{1+e^{prediction}},\;where$$

$$\begin{array}{l} prediction=-1.078+(1.309*loss\;of\;smell)+(0.481*fever)+(0.407*COVID\;at\;home)\\ \;+(0.338*shortness\;of\;breath)+(0.237*myalgia)+(0.153*cough)\\ \;+(0.035*nausea)+(0.033*gender[male])+(0.008*age)\\ \;-(0.441*sore\;throat)-(0.227*coryza)-(0.045*diarrhea) \end{array}$$ (2)

The probability of an individual be a positive case can be calculated by Eq 2, using the log-odds of the positive test occurring (prediction).

**Fig 3:**
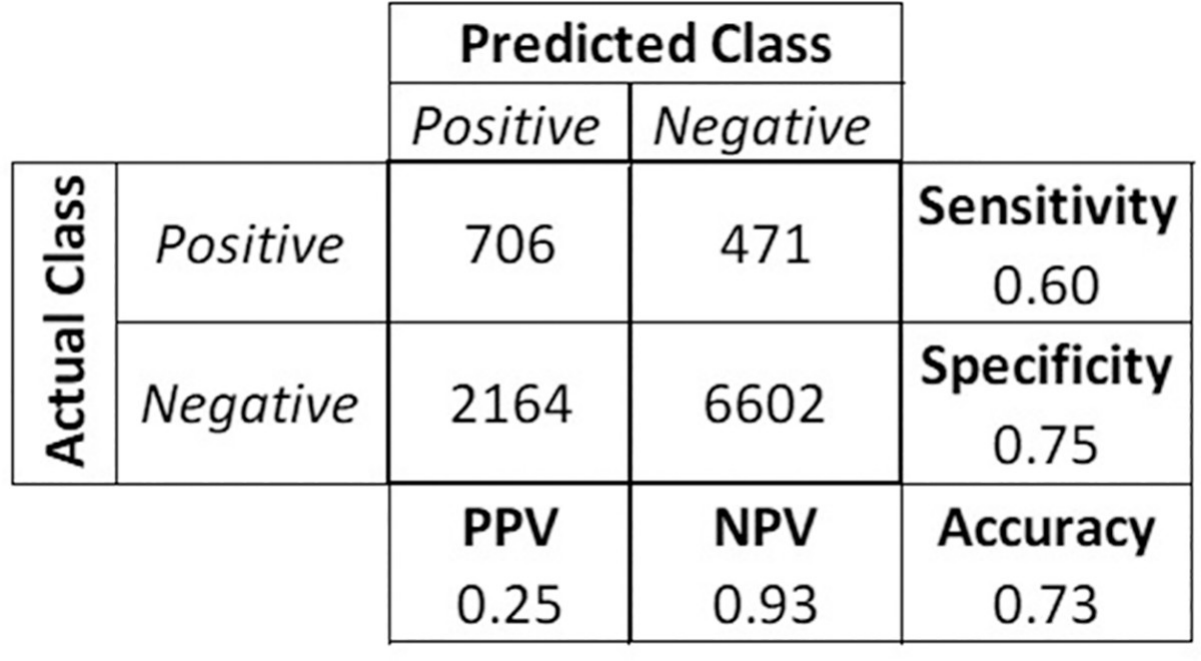
Confusion matrix and performance metrics of the final model.

Regarding the classification metrics (Fig 3), our model performed as follows: Sensitivity (Recall) = 0.60; Specificity = 0.75; PPV (Precision) = 0.25; NPV = 0.93; F1 = 0.35; MCC = 0.25; AUC = 0.68. Thus, evaluating the predicted probability values, we observed that our model correctly predicts almost all negative tests with only 7% of false-negative users among the predicted as negatives (NPV = 0.93).

The characteristics of the false-negative and false-positive cases predicted by our model can be seen in Table 2.

**Table 2:** Characteristics and symptoms of false-negative and false-positive users predicted from our model.

	False-negative	False-positive
**Participants, n (%) **	471	2,164
**Characteristics **		
Female, n (%)	258 (54·8)	1,356 (62·7)
Age (years), median [IQR]	42 [34–51]	42 [32–52]
Cohabitation—lives with a SARS-CoV-2 infected person, n (%)	179 (38·0)	1,464 (67·7)
**Self-reported symptoms, n (%) **		
Loss of smell	4 (0.8)	1,694 (78.3)
Fever	88 (18.7)	1,288 (59.5)
Myalgia	156 (33.1)	1,473 (68.1)
Cough	197 (41.8)	1,511 (69·8)
Nausea	43 (9.1)	429 (198)
Sore throat	173 (36.7)	1,062 (49.1)
Coryza	200 (42.5)	1,321 (61.0)
Diarrhea	88 (18.7)	731 (33.8)
Shortness of breath	2 (0.4)	32 (1.5)

We observed that most of the false-positive cases present the top-four predictors with the highest positive coefficients. Simultaneously, only four false negatives reported the loss of smell—the strongest predictor of a positive test. The probability density function and frequency of the model’s predicted values using a testing set, compared to the real (observed) values, can be seen in S2 Fig.

### SARS-CoV-2 risk areas in Rio de Janeiro

**Fig 4:**
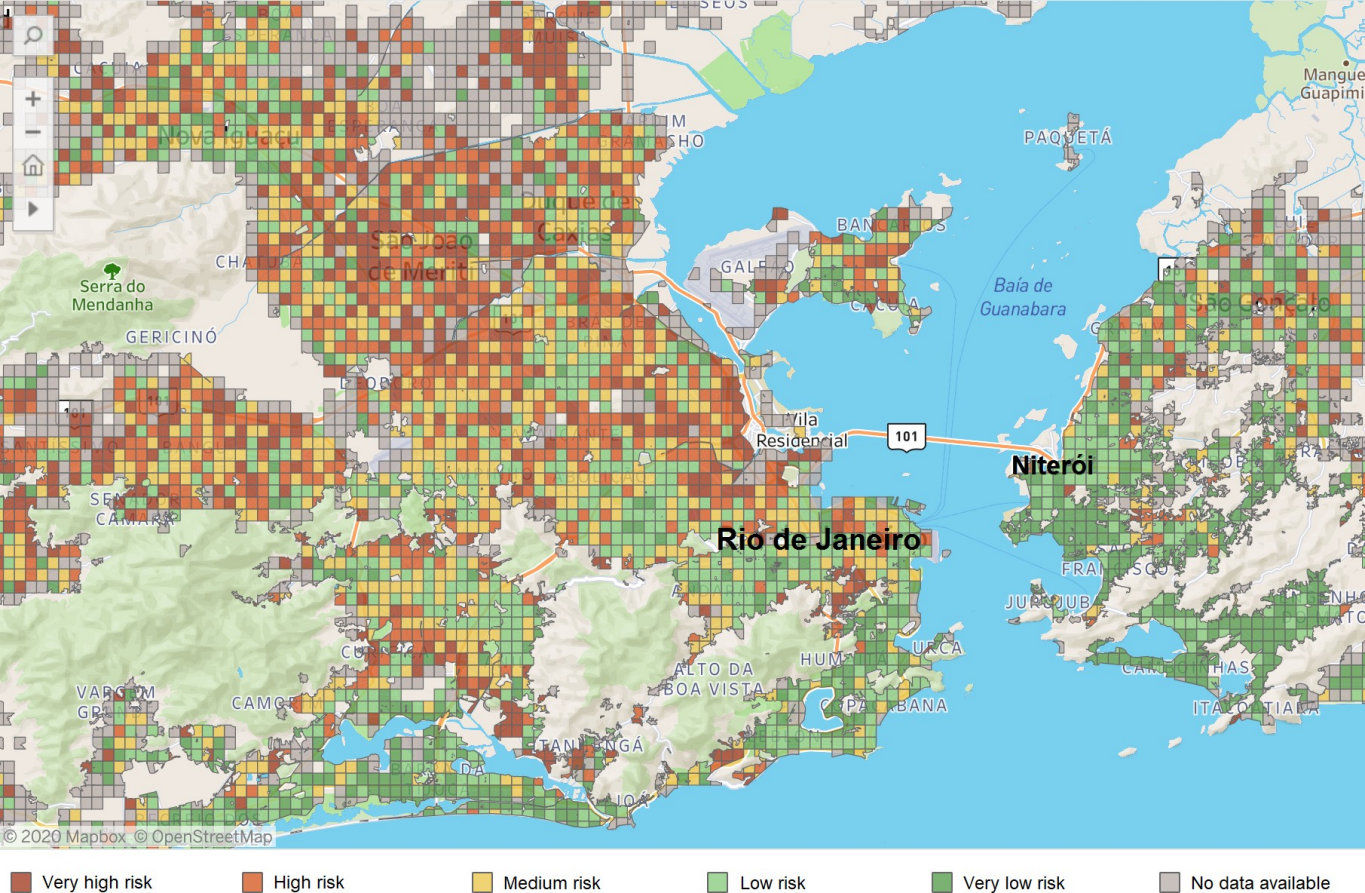
**SARS-CoV-2 infection risk map (grid) of Rio de Janeiro state, displaying the city of Rio de Janeiro (capital, left) and the city of Niteroi (right).** The grid risk considered the proportion of potential positive infection (observed test results + estimated from the prediction model) for each grid (400mx400m area). The risk groups were obtained as very low (<17%), low (≥17% and <33%), medium (≥33% and <48%), high (≥48 and <63%), and very high (≥63%). The map shows the distribution of risks as of June 10, 2020. Map created by *Dados do Bem* app using OpenStreetMap @ 2020Mapbox.

To optimize the testing strategy, we applied the predictive model (Eq 2) to the 287,714 individuals who registered in the app and were still untested for SARS-CoV-2. According to our model, 99,431 (34.5%) of these participants were classified as positive. We calculated the proportion of positive test results for each grid in Rio de Janeiro by Eq 1, visualizing the predicted SARS-CoV-2 infected cases (Fig 4). As of July 16, 2020, we observed that the southern (richer) areas in Rio de Janeiro presented lower proportions of potential positive participants than the northern (poorer) areas.

### External validation

The "Dados do Bem" app incorporated our final model on July 17, 2020, using it to prioritize users for testing in some Brazilian states. The external validation using data from Rio de Janeiro comprised 57,762 tests from August 01 to September 01, resulting in 18.1% positive results (10,466/57,762). If we consider data from June 15 to July 16 (before model implementation), we observed only 14.9% of positivity (5,296/35,626), thus indicating that the incorporated model increased the proportion of positive tests. The hypothesis test results showed a statistically significant difference between positive results proportion before and after the model implementation (p-value < 0.001 with a 95% confidence level).

## Discussion

Extensive testing programs for SARS-CoV-2 are, in general, not available in low- and middle-income countries, conferring the under-reporting of confirmed cases into a problem. A previous study estimated that only 9.2% of Brazilian cases are being notified [16]. Restricting tests hinder the monitoring of the epidemic progression, resource planning, and evaluation of the effectiveness of the control measures. Besides, it leads to false conclusions that the disease is under control.

Since it is impossible to test all individuals, some studies suggest that the combination of symptoms could be used as a screening tool to identify people with potential SARS-CoV-2 infection who could be selected for testing [4, 6, 17]. It can be useful for planning public policies and for preventing the spread of the pandemic. That said, our study used data on individual symptoms and demographics obtained from an app-based system, known as "*Dados do Bem*," to develop a model that predicts individuals with a higher probability of testing positive for SARS-CoV-2 infection.

Some works criticize the use of symptom-based screening strategies to quantify an individual likelihood of having COVID-19 due to the non-specific nature of some symptoms and the existence of co-infections with other respiratory viruses [18]. However, our results evidenced that such a strategy contributes to optimizing the overall testing strategy. Out of the 287,714 new users still not tested, our model estimated that the virus could infect 99,431 who, therefore, should be prioritized for testing. It reduced the need for extensive testing to only 34.5% of the registered untested users. This is undoubtedly beneficial as a public policy, especially in Brazil, a country with the second highest COVID-19 cases and one of the lowest test rates^1^.

Our model was incorporated into the app and used to select patients for testing. We chose the city of Rio de Janeiro to evaluate the benefit of using our model. Out of the 57,762 users selected according to the model, 18.1% were tested positive. This positivity rate is statistically significant compared to the observed positivity rate without a model (14.9%). It indicates that our model contributed to improve the test strategy and to select the users most likely to be positive in the current scenario. Hallal et al. [8] performed a SARS-CoV-2 antibody prevalence study analyzing 25,025 participants in the first survey (May 14–21) and 31,165 in the second (June 4–7) and showed that city-level prevalence in Rio de Janeiro was 2.4% (0.7–5.6%) in the first survey and 7.5% (4.5–11.7%) in the second one. Therefore, we note that the seroprevalence obtained throughout the utilization of the "*Dados do Bem*" app was higher than the city-level prevalence, thus leading to an improved testing strategy and helping achieve better use of scarce test resources.

In addition to forecasting the likelihood of each user acquiring the virus, our model also assesses these participants’ geographical distribution, being a source of information to build a risk map for Rio de Janeiro, as shown in Fig 4. The "*Dados do Bem*" app currently uses this map for categorizing risk areas, thus supporting decision-makers to identify areas with a higher risk of infection prevalence and accordingly refine testing and disease control strategies.

**Fig 5:**
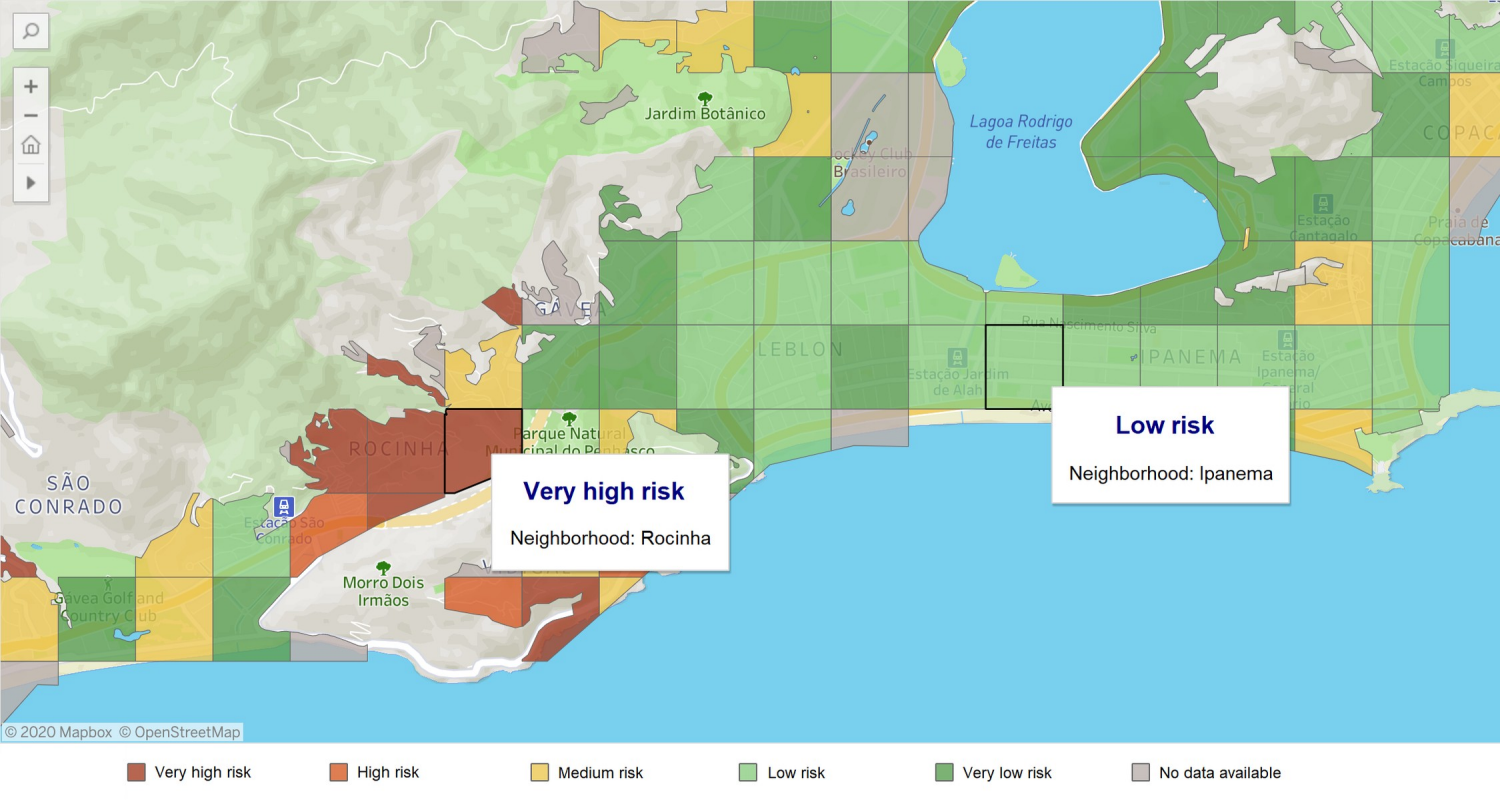
Risk map of two neighborhoods of Rio de Janeiro (Rocinha–very high risk and Ipanema–low risk). Map created by *Dados do Bem* app using OpenStreetMap @ 2020Mapbox.

The risk map analysis developed in this work is exemplified in Fig 5, which presents the risk map of the south zone of Rio de Janeiro. The chosen area includes both high-income neighborhoods (such as "*Ipanema*,*"* "*Leblon*,*"* and "*Gávea"*) and slums (such as "*Rocinha"*). The selected grid in Ipanema is classified as "low risk," which means that the proportion of positive tests in this grid was between 17% and 33%. The other selected grid is in Rocinha, which, although located less than three miles from Ipanema, is classified as a "very high risk" grid, meaning that the proportion of positive tests living in this grid was higher than 63%. Higher-risk areas in poor communities were also noted in other regions of the city (Fig 5). Many higher-risk grids were in the north zone of the city, where the most deprived communities are located ("*Complexo do Alemão*" and "*Complexo do São Carlos*,*"* for example). The presence of social inequalities in Brazil has been pointed out by previous studies [19–23], which noted that it could be associated with spreading the disease.

Regarding our results of the reported symptoms, loss of smell (anosmia) was the strongest indicator of SARS-CoV-2, followed by fever, shortness of breath, myalgia, cough, nausea, diarrhea, and coryza. The significant influence of loss of smell and cough is in line with previous studies carried out in high-income countries such as the US and UK [6, 24–26], and the influence of fever, myalgia, and nausea was pointed as significant in some studies [25, 26]. However, other authors noted it as not associated [6, 24]. Previous studies observed that sore throat, diarrhea, and shortness of breath are not significant predictors for the SARS-CoV-2 infection [6, 24–26].

Menni and colleagues [6, 17] used real-time tracking of self-reported symptoms similar to ours to predict potential SARS-CoV-2 infection in a cohort of individuals from the US and UK. This model was applied to compare the incidence in the UK regions. The authors noted that, in southern Wales, users reported symptoms that predicted, 5 to 7 days in advance, two spikes in the number of confirmed positive SARS-CoV-2 infection reported by public health authorities. The prediction models presented NPV of 0.75 and 0.87 in the UK (15,638 participants) and the US (2,763 participants). Compared to them, our best model obtained a competitive performance (NPV of 0.93).

Sebo and colleagues [26] studied a sample of 1,543 primary care patients tested in two laboratories in the Lyon area (France). They found that the two symptoms most strongly associated with a positive test were loss of taste (ageusia) and loss of smell. Combining these symptoms resulted in an even stronger association (i.e., the odds of having a positive test were six times greater than the odds of having a negative test). A recent literature review of studies analyzing the presence of loss of taste and smell in SARS-CoV-2 infected patients concluded that, from a total of 10,818 patients, 8,823 presented ageusia (81.6%) and 8,088 presented anosmia (74.8%) [27]. Our results reinforce the literature conclusions about the strong influence of loss of smell.

This study presents some limitations. First, the symptoms are self-reported. Hence, the participant may report apparent manifestations of the disease, which may not be precise as a physician’s physiological evaluation. Second, we could not know when a symptom appeared to indicate the disease’s stage at the testing moment. Third, a non-negligible number of false negatives may be present, considering the serological test’s sensitivity. However, identifying potential clusters and optimizing testing resources using a combination of self-reported symptoms is a viable strategy for many countries. A similar combination of symptoms can explain the SARS-CoV-2 infections in developed countries, such as the United Kingdom and the United States, and LMIC, such as Brazil. Fourth, we do not guarantee that the dataset represents the Brazilian population since our objective was not to perform an epidemiological study. Instead, we aimed to analyze the combination of self-reported symptoms from all users who registered in the city of Rio de Janeiro and obtained a test result (either positive or negative) until July 16, 2020.

## Conclusions

Our work used data regarding individual symptoms and demographics obtained from an app-based system to predict individuals with a higher probability of being infected by SARS-CoV-2. We developed a screening model and incorporated it into the app, aiming to prioritize users for testing. After applying the model, out of the 57,762 users selected, 18.1% were tested positive. This positivity rate was more significant than the one observed without a model (14.9%), which indicates that our model contributed to improve the test strategy and select the users most likely to be positive in the current scenario. Moreover, we developed a risk map derived from the model, which may help decision-makers locate regions with a higher risk of positive tests, allowing better testing and disease control policies.

## Supporting information

S1 Fig Pages of the COVID symptom tracker app. (TIF)

S2 Fig Probability density function and frequency of the predicted values by the model using a testing set, compared to the real (observed) values. The black vertical line corresponds to the cut-off of 0.5, and the colored dashed vertical lines correspond to the expected average probability for the group of negative (red) and positive (blue) groups.
(TIF)

S1 Table Performance of the ML models computed from the independent test set for each combination. (DOCX)

S1 File Selection criteria for the test. (DOCX)

S2 File Supplemental methods. Statistical analysis and machine learning methods. Predictive modeling. Definitions about the Balancing approaches.
(DOCX)
