# Supplementary figures and images for: App-based symptom tracking to optimize SARS-CoV-2 testing strategy using machine learning
Source: PLoS One. 2021 Mar 25;16(3):e0248920. doi: 10.1371/journal.pone.0248920 (PMC7993758; doi:10.1371/journal.pone.0248920)

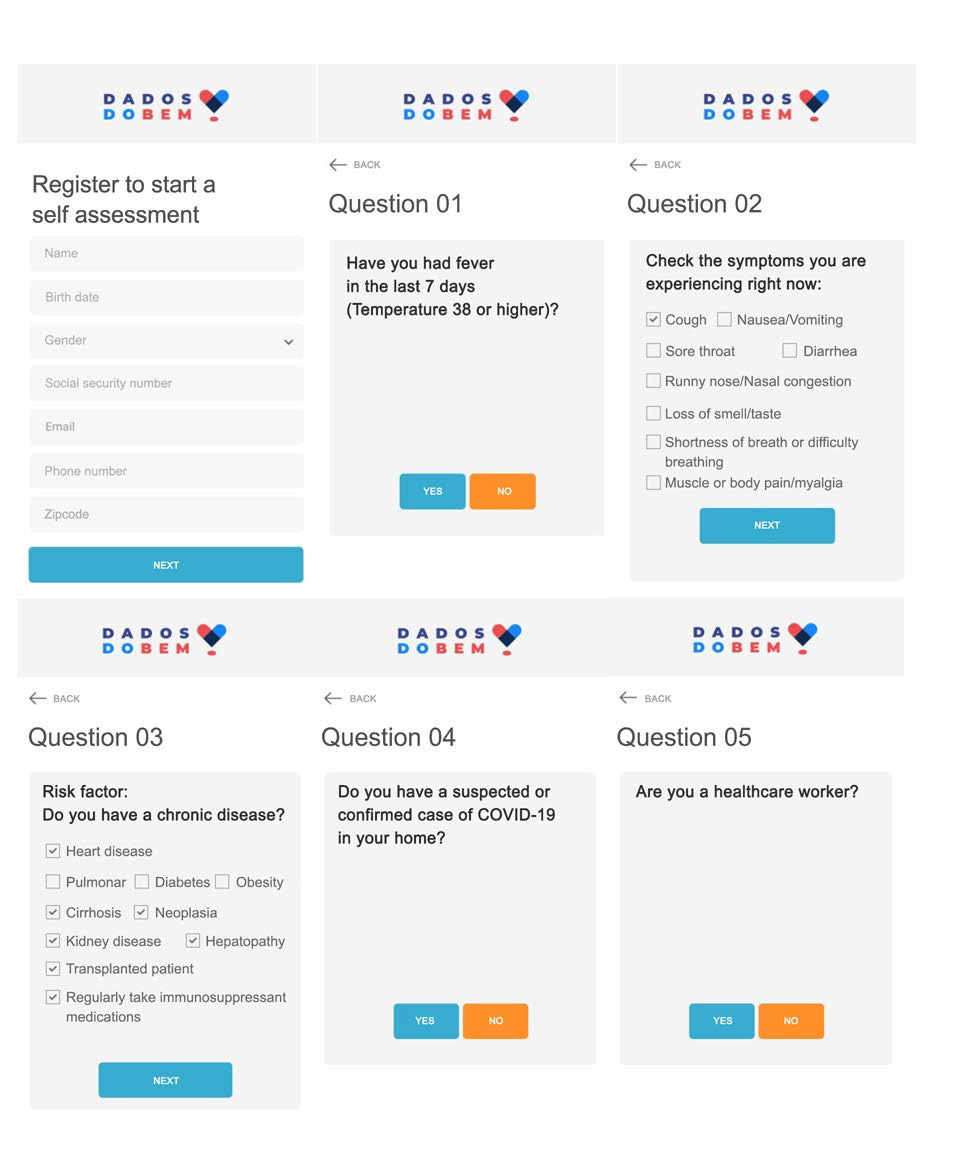

Supplement: S1 Fig — (TIF) [file pone.0248920.s001.tif]

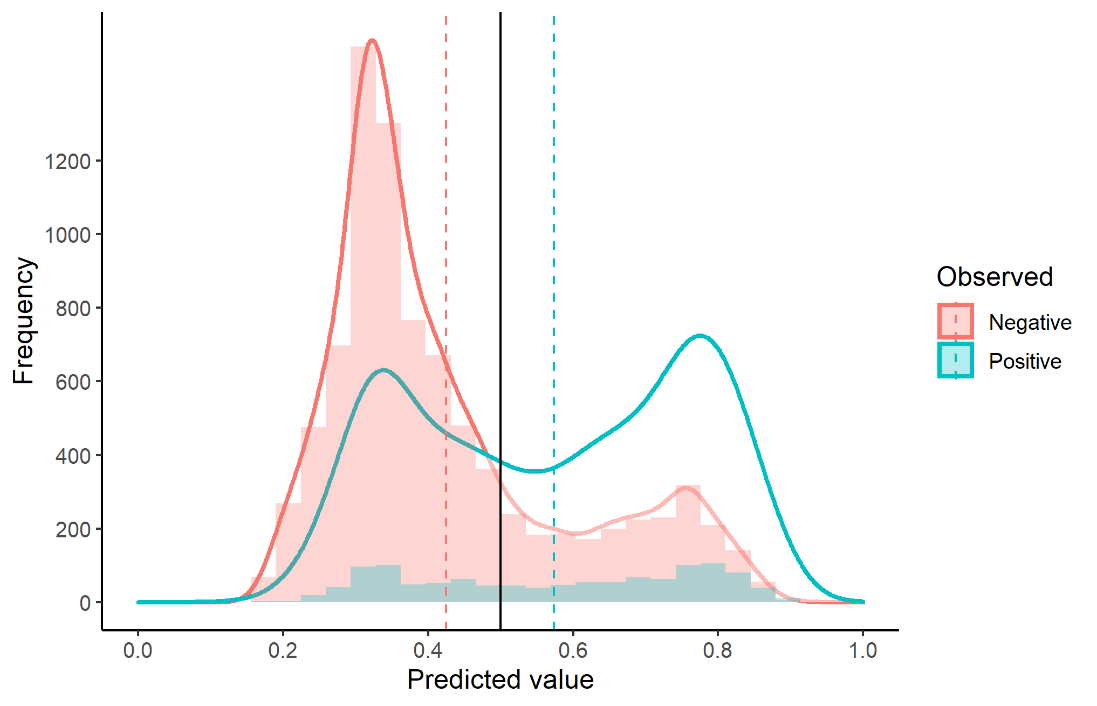

Supplement: S2 Fig — The black vertical line corresponds to the cut-off of 0.5, and the colored dashed vertical lines correspond to the expected average probability for the group of negative (red) and positive (blue) groups. (TIF) [file pone.0248920.s002.tif]
