# Supplementary material for: App-based symptom tracking to optimize SARS-CoV-2 testing strategy using machine learning
Source: PLoS One. 2021 Mar 25;16(3):e0248920. doi: 10.1371/journal.pone.0248920 (PMC7993758; doi:10.1371/journal.pone.0248920)
Supplement: S1 Table — (DOCX) [file pone.0248920.s003.docx]

**S1 Table. Performance of the ML models computed from the independent test set for each combination**

| **Method** | **Sampling Strategy** | **TN** | **FP** | **FN** | **TP** | **Accuracy** | **Kappa** | **Sensitivity** | **Specificity** | **PPV** | **NPV** | **Precision** | **Recall** | **F1** | **AUC** | **MCC** |
| --- | --- | --- | --- | --- | --- | --- | --- | --- | --- | --- | --- | --- | --- | --- | --- | --- |
| Decision Tree | null | 8766 | 0 | 1177 | 0 | 0.88 | 0.00 | 0.00 | 1.00 | NA | 0.88 | NA | 0.00 | NA | 0.50 | 0.00 |
| Decision Tree | down | 6408 | 2358 | 454 | 723 | 0.72 | 0.20 | 0.61 | 0.73 | 0.23 | 0.93 | 0.23 | 0.61 | 0.34 | 0.67 | 0.24 |
| Decision Tree | up | 6844 | 1922 | 583 | 594 | 0.75 | 0.19 | 0.50 | 0.78 | 0.24 | 0.92 | 0.24 | 0.50 | 0.32 | 0.64 | 0.21 |
| Decision Tree | smote | 7199 | 1567 | 587 | 590 | 0.78 | 0.24 | 0.50 | 0.82 | 0.27 | 0.92 | 0.27 | 0.50 | 0.35 | 0.66 | 0.25 |
| Decision Tree | rose | 8651 | 115 | 1084 | 93 | 0.88 | 0.10 | 0.08 | 0.99 | 0.45 | 0.89 | 0.45 | 0.08 | 0.13 | 0.53 | 0.15 |
| Logistic Regression | null | 8764 | 2 | 1173 | 4 | 0.88 | 0.01 | 0.00 | 1.00 | 0.67 | 0.88 | 0.67 | 0.00 | 0.01 | 0.50 | 0.04 |
| Logistic Regression | down | 6673 | 2093 | 487 | 690 | 0.74 | 0.22 | 0.59 | 0.76 | 0.25 | 0.93 | 0.25 | 0.59 | 0.35 | 0.67 | 0.25 |
| **Logistic Regression** | **up** | **6602** | **2164** | **471** | **706** | **0.73** | **0.22** | **0.60** | **0.75** | **0.25** | **0.93** | **0.25** | **0.60** | **0.35** | **0.68** | **0.25** |
| Logistic Regression | smote | 7028 | 1738 | 607 | 570 | 0.76 | 0.20 | 0.48 | 0.80 | 0.25 | 0.92 | 0.25 | 0.48 | 0.33 | 0.64 | 0.22 |
| Logistic Regression | rose | 6626 | 2140 | 481 | 696 | 0.74 | 0.22 | 0.59 | 0.76 | 0.25 | 0.93 | 0.25 | 0.59 | 0.35 | 0.67 | 0.25 |
| Naive Bayes | null | 8193 | 573 | 925 | 252 | 0.85 | 0.17 | 0.21 | 0.93 | 0.31 | 0.90 | 0.31 | 0.21 | 0.25 | 0.57 | 0.17 |
| Naive Bayes | down | 5945 | 2821 | 414 | 763 | 0.67 | 0.17 | 0.65 | 0.68 | 0.21 | 0.93 | 0.21 | 0.65 | 0.32 | 0.66 | 0.22 |
| Naive Bayes | up | 5984 | 2782 | 418 | 759 | 0.68 | 0.18 | 0.64 | 0.68 | 0.21 | 0.93 | 0.21 | 0.64 | 0.32 | 0.66 | 0.22 |
| Naive Bayes | smote | 6578 | 2188 | 559 | 618 | 0.72 | 0.17 | 0.53 | 0.75 | 0.22 | 0.92 | 0.22 | 0.53 | 0.31 | 0.64 | 0.20 |
| Naive Bayes | rose | 5930 | 2836 | 413 | 764 | 0.67 | 0.17 | 0.65 | 0.68 | 0.21 | 0.93 | 0.21 | 0.65 | 0.32 | 0.66 | 0.22 |
| Random Forest | null | 8766 | 0 | 1177 | 0 | 0.88 | 0.00 | 0.00 | 1.00 | NA | 0.88 | NA | 0.00 | NA | 0.50 | 0.00 |
| Random Forest | down | 6433 | 2333 | 450 | 727 | 0.72 | 0.21 | 0.62 | 0.73 | 0.24 | 0.93 | 0.24 | 0.62 | 0.34 | 0.68 | 0.25 |
| Random Forest | up | 6623 | 2143 | 470 | 707 | 0.74 | 0.22 | 0.60 | 0.76 | 0.25 | 0.93 | 0.25 | 0.60 | 0.35 | 0.68 | 0.25 |
| Random Forest | smote | 6759 | 2007 | 539 | 638 | 0.74 | 0.20 | 0.54 | 0.77 | 0.24 | 0.93 | 0.24 | 0.54 | 0.33 | 0.66 | 0.23 |
| Random Forest | rose | 6569 | 2197 | 472 | 705 | 0.73 | 0.21 | 0.60 | 0.75 | 0.24 | 0.93 | 0.24 | 0.60 | 0.35 | 0.67 | 0.25 |
| Gradient Boosting | null | 8751 | 15 | 1147 | 30 | 0.88 | 0.04 | 0.03 | 1.00 | 0.67 | 0.88 | 0.67 | 0.03 | 0.05 | 0.51 | 0.11 |
| Gradient Boosting | down | 6519 | 2247 | 458 | 719 | 0.73 | 0.21 | 0.61 | 0.74 | 0.24 | 0.93 | 0.24 | 0.61 | 0.35 | 0.68 | 0.25 |
| Gradient Boosting | up | 6515 | 2251 | 449 | 728 | 0.73 | 0.22 | 0.62 | 0.74 | 0.24 | 0.94 | 0.24 | 0.62 | 0.35 | 0.68 | 0.26 |
| Gradient Boosting | smote | 7284 | 1482 | 611 | 566 | 0.79 | 0.24 | 0.48 | 0.83 | 0.28 | 0.92 | 0.28 | 0.48 | 0.35 | 0.66 | 0.25 |
| Gradient Boosting | rose | 8754 | 12 | 1166 | 11 | 0.88 | 0.01 | 0.01 | 1.00 | 0.48 | 0.88 | 0.48 | 0.01 | 0.02 | 0.50 | 0.05 |
| Legend: TN = True Negative; FP = False Positive; FN = False Negative; TP = True Positive; PPV = Positive Predictive Value; NPV = Negative Predictive Value; AUC = Area Under the Curve; MCC = Matthews Correlation Coefficient. | | | | | | | | | | | | | | | | |
