# Supplementary material for: App-based symptom tracking to optimize SARS-CoV-2 testing strategy using machine learning
Source: PLoS One. 2021 Mar 25;16(3):e0248920. doi: 10.1371/journal.pone.0248920 (PMC7993758; doi:10.1371/journal.pone.0248920)
Supplement: S1 File — (DOCX) [file pone.0248920.s004.docx]

**S1 File. Selection Criteria for the test**

Group A (Highest Priority): Individuals that a previously positively tested participant indicated;

Group B: Individuals who were either:

- health professionals; or
- over 60 years old AND had at least ONE risk factor; or
- over 60 years old AND were in contact at home with someone who was positively tested.

Group C: Individuals who either:

- were in contact at home with someone who was positively tested AND presented TWO or more symptoms; or
- presented TWO or more symptoms AND had at least ONE risk factor.

Group D (Lowest Priority): Individuals who

- presented THREE or more symptoms; or
- were in contact at home with someone who was positively tested.
