# Supplementary material for: App-based symptom tracking to optimize SARS-CoV-2 testing strategy using machine learning
Source: PLoS One. 2021 Mar 25;16(3):e0248920. doi: 10.1371/journal.pone.0248920 (PMC7993758; doi:10.1371/journal.pone.0248920)
Supplement: S2 File — Statistical analysis and machine learning methods. Predictive modeling. Definitions about the Balancing approaches. (DOCX) [file pone.0248920.s005.docx]

**S2 File. Supplemental Methods**

**Statistical analysis and machine learning methods.**

Our study aimed to obtain a model for estimating the test result for SARS-CoV-2 infection based on the patient's characteristics and symptoms. Since the response variable is a binary outcome (positive or negative), we identify a supervised learning problem of classification.

We split our dataset into training and testing sets in our learning strategy, using the 80%/20% ratio using random sampling. With the training set, we obtained the best model parameters using the 5-fold cross-fold validation, in which each model set is evaluated in 1 part of the dataset. In contrast, the other four are used to estimate the model. We chose the best parameters were the ones that maximized the Area Under the Receiving Operating Characteristic curve (AUROC or AUC), which is a trade-off between the True Positive Rate (TPR) and the False Positive Rate (FPR).

$$TPR=\frac{True Positive}{True Positive+False Negative}$$

$$FRP=1-True Negative Rate=1- \frac{True Negative}{True Negative+False Positive}$$

We considered data balancing techniques during the training step to account for the response variable's possible imbalance. For each combination of model and data balancing method, we obtained the AUC. Finally, we evaluated the models' performance in the testing set chose the one with the highest Matthew Correlation Coefficient (MCC).

$$Matthews Correlation Coefficient \left( \mathrm{MCC} \right)= \frac{TP x TN-FP x FN}{\sqrt{\left( TP+FP \right)\left( TP+FN \right)\left( TN+FP \right)\left( TN+FN \right)}}$$

The models were implemented with the *caret* package in R 3.6.3, which provides a general framework to train and evaluated models. We provide codes used for our analysis in a Github repository (https://github.com/noispuc/Dantas_etal_PLOSOne_App-based-symptom).

**Predictive modeling**

We considered five different statistical and machine learning methods: the logistic regression, the Naïve Bayes classifier, Decision Trees, Random Forest, and Gradient Boosting Trees. Those methods are briefly described in the following. Each model was implemented using the *caret* package from R.

A) In the Logistic regression model, we used the stepwise selection approach to identify the subset of variables (symptoms and patient's characteristics) that best predicts the response variable (positive or negative test result) (EFROYMSON, 1960). In our experiments, the best logistic regression model is the one that maximizes the Area Under the Receiving Operating Characteristic curve (AUC) during the stepwise process. Since the logistic regression model provides probabilities as the predicted response, we considered a cut-off of 0.5 to obtain classes: The chosen cut-off point for predicted values was 50%, i.e., participants with a probability higher than 50% were classified as "positive" otherwise "negative." Considering Y, the response variable, and X, the set of predictors, the model is:

$$Prediction=logit\left( Y \right)=BX$$

, where B is the vector of coefficients for each predictor variable.

The estimated probability is obtained as $Probability= \frac{e^{Prediction}}{1+e^{Prediction}}$

B) The Naive Bayes classifier (NB) is based on Bayes' rule (FRIEDMAN; GEIGER; GOLDSZMIDT, 1997), in which the classification is based on the conditional probability of each class (e.g., positive or negative) given the predictors, assuming that they are independent (PERIWAL et al., 2011). The predicted class (or response) is obtained as the one with maximum posterior probability. To define the best set of this model's parameters, we considered the set that maximizes the AUC.

C) Decision Trees (DT) are tree-based methods used to estimate a certain response given a set of features (predictors). They use a recursive splitting mechanism to grow a tree of features. Each split in the predictors' space is chosen to maximize information gain, minimize entropy or minimize the classification error (PATEL; UPADHYAY, 2012). In our study, we selected the DT with the highest AUC.

D) The Random Forests (RF) method is an ensemble of Decision Trees aggregated to achieve a better performance than a single tree model. This method estimates "simple" decision trees by re-sampling the dataset and the feature space and obtaining their predicted response. Then, the classification aggregates the predicted responses from the trees (BREIMAN, 2001). We selected the best RF setting as the one that maximizes the AUC.

E) Gradient Boosting Trees (GBT) (FRIEDMAN, 2002) is an ensemble of decision trees, like the random forest. However, instead of estimating trees based on the dataset's re-sampled versions, each new tree is obtained from previous trees' prediction errors, thus improving the classifier. We selected the best GBT setting as the one that maximizes the AUC.

BREIMAN, Leo. Random forests. Machine learning, v. 45, n. 1, p. 5-32, 2001.

EFROYMSON, M. A. Multiple regression analysis. Mathematical methods for digital computers, p. 191-203, 1960.

FRIEDMAN, Jerome H. Stochastic gradient boosting. Computational statistics & data analysis, v. 38, n. 4, p. 367-378, 2002.

FRIEDMAN, Nir; GEIGER, Dan; GOLDSZMIDT, Moises. Bayesian network classifiers. Machine learning, v. 29, n. 2-3, p. 131-163, 1997.

KUHN, Max et al. Applied predictive modeling. New York: Springer, 2013.

PATEL, Nikita; UPADHYAY, Saurabh. Study of various decision tree pruning methods with their empirical comparison in WEKA. International journal of computer applications, v. 60, n. 12, 2012.

PERIWAL, Vinita et al. Predictive models for anti-tubercular molecules using machine learning on high-throughput biological screening datasets. BMC research notes, v. 4, n. 1, p. 504, 2011.

**Definitions about the Balancing approaches**

The sampling methods consist of modifying an imbalanced dataset to provide a balanced distribution (VANHOEYVELD; MARTENS, 2018; YANG; GAO, 2013) and are considered data level solutions. These techniques generally consist of oversampling (up-sampling) the minority class, under-sampling (down-sampling) the majority class, or a combination.

A) Down-sampling occurs when some majority class instances are not considered, creating a sample with the same number of cases as the minority class (CRONE; FINLAY, 2012). Up-sampling is a method that aims to balance class distribution by randomly replicating instances of the minority class (BATISTA; PRATI; MONARD, 2004). More advanced techniques, such as SMOTE and ROSE, introduce synthetic samples in which the classifier creates more significant and less specific decision regions.

B) Chawla et al. (2002) proposed the Synthetic Minority Over-sampling Technique (SMOTE). It is an over-sampling method that aims to create a new instance of the minority class by interpolating several examples (of the minority class) that lie together. The SMOTE algorithm selects k-nearest neighbors for each instance in the minority class (YANG; GAO, 2013).

C) Lunardon et al. (2014) proposed the Random OverSampling Examples (ROSE) technique. It is based on a smoothed bootstrap form of re-sampling from data, which combines oversampling and under-sampling techniques by generating a larger sample of data. ROSE allows the generation of some clones of the observed data without producing ties, obtaining synthetic examples from an estimate of the (conditional) density underlying the data.

BATISTA, Gustavo EAPA; PRATI, Ronaldo C.; MONARD, Maria Carolina. A study of the behavior of several methods for balancing machine learning training data. ACM SIGKDD explorations newsletter, v. 6, n. 1, p. 20-29, 2004.

CARUANA, Rich et al. Intelligible models for healthcare: Predicting pneumonia risk and hospital 30-day readmission. In: Proceedings of the 21st ACM SIGKDD international conference on knowledge discovery and data mining. 2015. p. 1721-1730.

CHAWLA, Nitesh V. et al. SMOTE: synthetic minority over-sampling technique. Journal of artificial intelligence research, v. 16, p. 321-357, 2002.

CRONE, Sven F.; FINLAY, Steven. Instance sampling in credit scoring: An empirical study of sample size and balancing. International Journal of Forecasting, v. 28, n. 1, p. 224-238, 2012.

LUNARDON, Nicola; MENARDI, Giovanna; TORELLI, Nicola. ROSE: A Package for Binary Imbalanced Learning. R Journal, v. 6, n. 1, 2014.

VANHOEYVELD, Jellis; MARTENS, David. Imbalanced classification in sparse and large behaviour datasets. Data Mining and Knowledge Discovery, v. 32, n. 1, p. 25-82, 2018.

YANG, Zeping; GAO, Daqi. Classification for imbalanced and overlapping classes using outlier detection and sampling techniques. Applied Mathematics & Information Sciences, v. 7, n. 1, p. 375-381, 2013.
